# *Supt16* Haploinsufficiency Impairs PI3K/AKT/mTOR/Autophagy Pathway in Human Pluripotent Stem Cells Derived Neural Stem Cells

**DOI:** 10.3390/ijms24033035

**Published:** 2023-02-03

**Authors:** Junwen Wang, Ziyi Wang, Limeng Dai, Xintong Zhu, Xingying Guan, Junyi Wang, Jia Li, Mao Zhang, Yun Bai, Hong Guo

**Affiliations:** Department of Medical Genetics, College of Basic Medical Science, Army Medical University (Third Military Medical University), Chongqing 400038, China

**Keywords:** neurodevelopmental disorders (NDDs), *Supt16*, human neural stem cells (hNSCs), proliferation, autophagy

## Abstract

The maintenance of neural stem cells (NSCs) plays a critical role in neurodevelopment and has been implicated in neurodevelopmental disorders (NDDs). However, the underlying mechanisms linking defective human neural stem cell self-renewal to NDDs remain undetermined. Our previous study found that *Supt16* haploinsufficiency causes cognitive and social behavior deficits by disrupting the stemness maintenance of NSCs in mice. However, its effects and underlying mechanisms have not been elucidated in human neural stem cells (hNSCs). Here, we generated *Supt16*^+/−^ induced pluripotent stem cells (iPSCs) and induced them into hNSCs. The results revealed that *Supt16* heterozygous hNSCs exhibit impaired proliferation, cell cycle arrest, and increased apoptosis. As the RNA-seq analysis showed, *Supt16* haploinsufficiency inhibited the PI3K/AKT/mTOR pathway, leading to rising autophagy, and further resulted in the dysregulated expression of multiple proteins related to cell proliferation and apoptotic process. Furthermore, the suppression of *Supt16* heterozygous hNSC self-renewal caused by autophagy activation could be rescued by MHY1485 treatment or reproduced in rapamycin-treated hNSCs. Thus, our results showed that *Supt16* was essential for hNSC self-renewal and its haploinsufficiency led to cell cycle arrest, impaired cell proliferation, and increased apoptosis of hNSCs by regulating the PI3K/AKT/mTOR/autophagy pathway. These provided a new insight to understand the causality between the *Supt16* heterozygous NSCs and NDDs in humans.

## 1. Introduction

Neurodevelopmental disorders (NDDs) are complex disorders with heterogeneous etiologies, characterized by a defect in cognitive, emotional, and motor developmental milestones. They constitute serious health and financial problems in our society, affecting >3% of children worldwide [1]. NDDs include autism spectrum disorder (ASD), intellectual disability (ID), attention deficit hyperactivity disorder, and epilepsy [2,3]. During development, neural stem cells (NSCs) lining the neural tube generate the entire nervous system [4]. Research has found that NSCs originate from embryos and adults and express neural-specific markers, such as SOX2 and PAX6 genes. The neuroepithelial cells of the neural tube give rise to NSCs and radial glia (RG) during late embryogenesis. In adults, RG produce the astrocyte-like NSCs in the subventricular zone (SVZ) and subgranular zone (SGZ) [5]. Numerous studies have revealed that NSCs undergo both symmetric divisions to expand the NSC pool and asymmetric divisions to produce intermediate progenitors and the differentiated cell types arising from NSCs in a temporally defined sequence, including neurons, astrocytes, and oligodendrocytes [6]. Maintenance of the NSC pool by self-renewal and committed differentiation are precisely regulated, essential for proper nervous system development. Thus, studying the underlying regulation mechanisms controlling NSC self-renewal was necessary to understand both the neural development and NDDs of human.

In recent years, several pieces of evidence have suggested that proliferation and differentiation in NSCs are a major convergence point of NDDs [7]. More and more studies have found that the abnormal proliferation of NSCs is a substantial etiology of NDDs [8,9]. It has been reported that NSC maintenance is an essential factor for normal neurodevelopment and excessive or defective proliferation of NSCs during the embryonic stage can lead to NDDs in animal models [8,10]. Moreover, studies have shown that the defective proliferation of neural stem/progenitor cells leads to microcephaly in mice [11,12]. Increased proliferation of the neuroepithelium during neural tube closure results in exencephaly [13,14]. Similar to the studies in animal models, there has been much convincing evidence about Huntington’s disease (HD) in the human brain exhibiting greater neural progenitor cell (NPC) proliferation, proportional to the severity of the gene defect [15]. Additionally, fewer proliferating cells in the SVZ human brain were observed during postmortem [16]. From these investigations, it is evident that the self-renewal of NSCs plays a critical role in the neural development of mammals. However, the many underlying genetic causes and the molecular mechanism mediating human neural stem cell (hNSC) self-renewal remain unclear.

Autophagy is a process of cell self-digestion through the lysosomal pathway, which plays a vital role in maintaining intracellular homeostasis and cytoprotection [17]. Its activity can be activated by the activator rapamycin [18] or inhibited by the inhibitor MHY1485 [19]. Numerous studies in cell and animal models have shown that autophagy has diverse physiological functions in different cell types and tissues [20]. Moreover, the physiological levels of autophagy have been reported as protective for neurodevelopment and its dysregulation leads to NDDs [21]. Furthermore, it was reported that the social and behavioral impairments in autophagy-deficient mice are analogous to those in ASD patients [22]. The inhibiting autophagy in mice results in microcephaly due to the impaired development of the main forebrain commissures and axonal connectivity [23]. In addition, postnatal spine pruning defects and ASD-like social behaviors were observed in mice with defective autophagy; the autophagy activator rapamycin can correct the ASD-like behavioral and spine pruning defects [24]. Consistent with this investigation, a plethora of studies have found that rapamycin can rescue the phenotypes of NDDs caused by autophagy defects [18,25,26]. Conversely, excitatory synapses with autophagosome accumulation show lower synaptic densities, less GRIA/AMPA receptor-mediated transmission, and worse neural network activity [27]. Autophagy plays an essential role in neurodevelopment and synaptic function and its deficits lead to NDDs. However, the underlying mechanism between autophagy and NDDs still needs to be further investigated, especially the effects of autophagy’s overactivation on neurodevelopment.

The *Supt16* gene encodes the large subunit of facilitates chromatin transcription (FACT), playing a vital role in the manipulation of nucleosomes [28,29,30]. It is highly expressed in the early stage of embryonic development. It has been found that the *Supt16* gene can directly regulate the Wnt pathway [31], Meanwhile, Shen, Z. et al. suggested that SPT16 cooperates with OCT4 to enhance the expression of stemness genes in mouse embryonic stem cells (mESCs) [32]. De novo *Supt16H* missense variants have been identified in neurodevelopmental disorder patients, resulting in autistic features, minor dysmorphic features, intellectual disability, and seizures [33]. Our previous article explored the pathogenic mechanism of the *Supt16* gene in a mouse model. The results showed that early mouse embryos were fatally affected by homozygous *Supt16* knockout and *Supt16* haploinsufficient mice exhibited abnormal cognitive and social behavior accompanied by neuronal reduction and abnormalities in the number and size of dendritic spines. Furthermore, *Supt16* heterozygous mouse neural stem cells (mNSCs) showed impaired proliferation, G1/S cell cycle arrest, and increased apoptosis. Mechanistically, *Supt16* haploinsufficiency inhibited the activity of the MAPK pathway. Our previous investigation has already demonstrated that *Supt16* haploinsufficiency disrupts the stemness of mNSCs by inhibiting the MAPK signal pathway in mice [34]. However, our study has some shortcomings; the results obtained in animal models cannot be a complete substitute for studies in human models, and whether there is a pathogenic mechanism of *Supt16* haploinsufficiency in human models remains to be further explored. Simultaneously, the effects of *Supt16* haploinsufficiency in hNSCs and the underlying mechanisms remain unclear.

Here, we used CRISPR/Cas9 technology to generate *Supt16*^+/−^ iPSCs and induced these to hNSCs exhibiting defects in self-renewal capacity compared to wild-type, which was accompanied by G1/S cell cycle arrest and increased hNSCs apoptosis. Mechanistically, *Supt16* haploinsufficiency changed the pattern of gene expression associated with the PI3K/AKT signaling pathway, mTOR pathway, and autophagy. Meanwhile, the activation of autophagy modulated by PI3K/AKT/mTOR pathway inhibition was also observed by WB analysis. These findings are consistent with previous studies showing that the mTOR signaling pathway is a pivotally negative regulator of autophagy [35,36]. Further studies also revealed that the reduced expression of PAX6 and SOX2 proteins induced by autophagy activation was detected in *Supt16* haploinsufficient hNSCs. Moreover, impaired hNSCs’ self-renewal ability caused by *Supt16* haploinsufficiency was reproduced in rapamycin-treated hNSCs, and MHY1485 treatment rescued the damaged *Supt16*^+/−^ hNSCs’ self-renewal ability. Ultimately, we demonstrated that *Supt16* haploinsufficiency disrupted hNSC self-renewal capacity via PI3K/AKT/mTOR pathway inhibition, raising autophagy.

## 2. Results

### 2.1. Supt16 Haploinsufficiency Disrupts the Self-Renewal of Human iPSCs Derived NSCs

**Figure 1:**
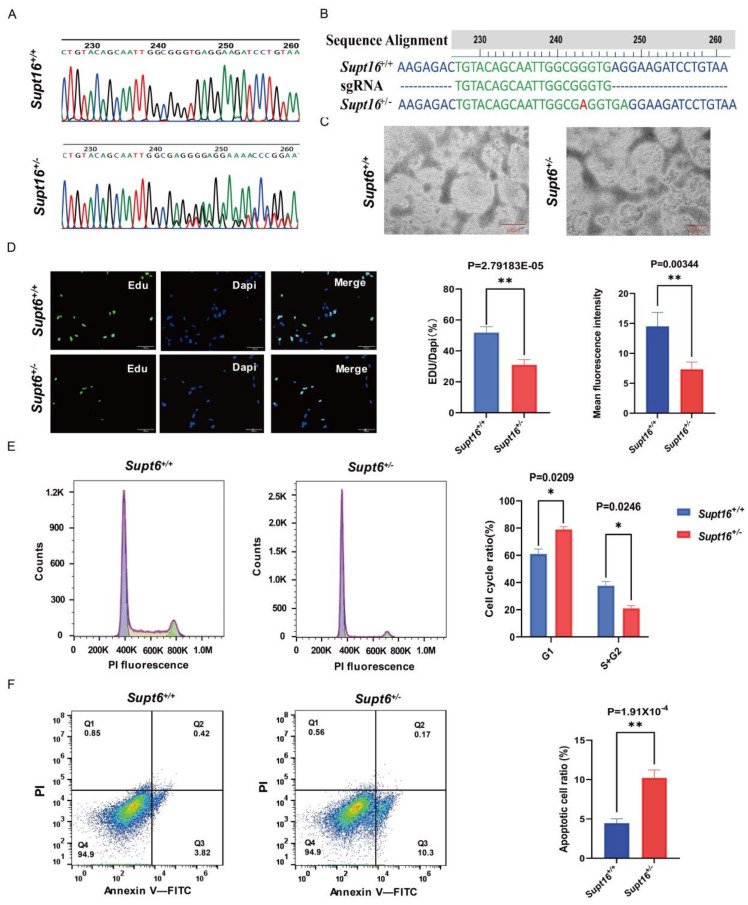
*Supt16* haploinsufficiency disrupts the self−renewal of human iPSC−derived NSCs. (**A**) Sanger sequencing of *Supt16* heterozygous hNSCs clone after CRISPR/Cas9−mediated. (**B**) *Supt16*^+/−^ hNSCs exhibit a point mutation in the form of an adenine nucleotide insertion (red hyphen). (**C**) Representative pictures of neural rosettes derived from *Supt16*^+/−^ haploinsufficiency and wild-type iPSCs; scale bar, 100 μm. (**D**) Representative immunofluorescence images of EdU incorporation assay (*n* = 3 samples per genotype. Green: EdU−positive cells. Blue: DAPI. Scale bar, 100 μm). (**E**) Representative flow cytometry analysis picture of hNSCs cell cycle (left). Quantitative analysis of hNSCs cell cycle distribution (right) (*n* = 3 samples of each genotype). (**F**) Representative flow cytometry analysis picture and quantification of hNSCs apoptotic rate (*n* = 3 samples of each genotype). The error bars represented the mean ± SD and the significance level was calculated by Student’s t−test (two−tailed, equal variance) (ns means not statistically significant, * *p* < 0.05, ** *p* < 0.01).

In our previous studies, we demonstrated that *Supt16* haploinsufficiency disturbs the neural stem cell proliferative capacity in the mouse brain SVZ [34]. However, whether *Supt16* haploinsufficiency has the same effects on hNSCs derived from iPSCs needs to be further investigated. To obtain the *Supt16* haploinsufficient hNSCs and analyze the function of SPT16 in hNSC self-renewal capacity, we generated *Supt16*^+/−^ iPSCs by using the CRISPR/Cas9 editing system. Guide RNAs (gRNAs) were designed to target the exon 1 of the *Supt16* gene. As revealed by Sanger sequencing around the CRISPR targeting site (Figure 1A and Figure S1A), we successfully obtained the CRISPR/Cas9 edited iPSC clones. After analyzing the Sanger sequencing results, we found that an adenine nucleotide (A) was inserted into the sgRNA target locus (g.157_158ins A) (Figure 1B). The results from WB analysis also revealed that the SPT16 protein level was significantly reduced in *Supt16*^+/−^ iPSCs (Figure S1B). Next, both *Supt16*^+/+^ and *Supt16*^+/−^ iPSCs were directly subjected to neural stem cell differentiation using an induction medium; the neural rosettes appeared on the sixth day (Figure 1C). Rosettes were then digested into single cells and cultured for two days before starting the experiment. *Supt16* haploinsufficient hNSCs formed smaller cell spheres during the culture process as results showed (Figure S1C), indicating the affected proliferation ability of hNSCs. Furthermore, the EdU incorporation assay was performed to investigate the proliferation of hNSCs. We found a notable difference in EdU cell density between the experimental and control groups, confirming that the proliferation of *Supt16*^+/−^ hNSCs was significantly decreased compared with the control group (Figure 1D). Flow cytometry with propidium iodide (PI) staining was performed to study whether *Supt16* haploinsufficiency affects the cell cycle. The results revealed that *Supt16* heterozygous hNSCs show sufficient G1/S cell cycle arrest compared to the control hNSCs (Figure 1E). The above results showed that *Supt16* plays a vital role in the maintenance of hNSC self-renewal by affecting proliferation and cell cycle arrest. In addition, the apoptotic rate was measured in hNSCs by flow cytometry with Annexin V-FITC and PI staining; the number of early apoptotic cells in the experimental group were more than that in the control group (Figure 1F). These investigations suggested that *Supt16* haploinsufficiency impairs hNSC self-renewal capacity by disrupting proliferation, blocking the cell cycle, and promoting apoptosis.

### 2.2. Supt16 Haploinsufficiency Impaired hNSCs Self-Renewal by Activating PI3K/AKT/mTOR-Mediated Autophagy

**Figure 2:**
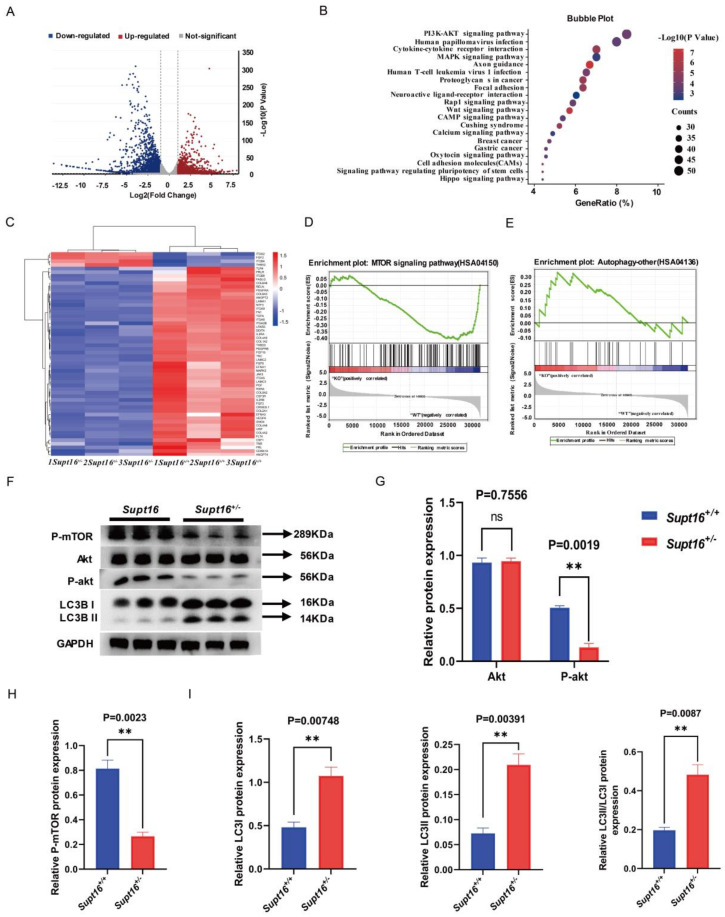
*Supt16* haploinsufficiency impaired hNSC self−renewal by activating PI3K/AKT/mTOR−mediated autophagy. (**A**) Volcano plots showed all differential expression genes detected by DESeq2 in *Supt16* heterozygous hNSCs. Each point represents an individual gene, of which 1629 genes are upregulated and 1976 genes are downregulated (p−value < 0.05; |log2foldchange| > 1). *n* = 3. (**B**) KEGG pathway analysis found that differential expression genes in *Supt16*^+/−^ hNSCs are enriched in the PI3K/AKT signaling pathway. (**C**) Heatmap analysis of PI3K/AKT signaling pathway−related differential expression genes enriched in KEGG analysis. (**D**) GSEA plot of differentially expressed genes for the list of mTOR signaling pathway genes (NES, normalized enrichment score; FDR, false discovery rate. NES = −1.79, FDR = 0.055). (**E**) GSEA analysis showing the expression pattern of autophagy−related genes in WT and *Supt16*^+/−^ hNSCs (NES, normalized enrichment score; FDR, false discovery rate. NES = 1.358, FDR = 0.254). (**F**) Western blot analysis showing AKT, P−akt, P−mTOR, and LC3 expression in hNSCs treated with different treatments (*n* = 3 samples per genotype). (**G**) Quantification analysis of AKT and P−akt protein relative to GAPDH. Data are means ± standard deviation of three independent experiments. (**H**) The P−mTOR expression was calculated relative to GAPDH. Data are means ± standard deviation of three independent experiments. (**I**) Quantification of LC3 protein relative to GAPDH. Data are means ± standard deviation of three independent experiments. The error bars represented the mean ± SD and the significance level was calculated by Student’s t-test (two-tailed, equal variance) (ns means not statistically significant, ** *p* < 0.01).

Given that *Supt16* is an essential chromatin regulator that contributes to DNA transcription regulation, we studied whether changes to gene expression in *Supt16* heterozygous hNSCs might underlie the impaired hNSCs’ self-renewal capacity phenotypes. RNA-seq was performed to explore the molecular mechanism underlying *Supt16* that modulates hNSC self-renewal. The results revealed that a total of 3605 differential expression genes (DEGs) (log2 fold change < −1 and *p* < 0.05) were identified in *Supt16*^+/−^ hNSCs compared with control littermates in RNA-seq data. An average 1629 genes were upregulated and 1976 genes were downregulated (Figure 2A). Kyoto encyclopedia of genes and genomes (KEGG) analysis of these differential expression genes showed enrichment in the PI3K/AKT pathway and heatmap analysis showed that the expression of genes associated with the PI3K/AKT signaling pathway enriched by KEGG was mainly downregulated, indicating that PI3K/AKT pathway activity was inhibited (Figure 2B,C). To further study the effect of *Supt16* gene heterozygous knockout on gene expression, we performed gene set enrichment analysis (GSEA) on the differential expression genes. The investigation showed that mTOR pathway activity was significantly reduced in the *Supt16*^+/−^ hNSCs compared with control hNSCs (Figure 2D), consistent with the observed result associated with the PI3K/AKT pathway. The expression levels of different genes associated with the mTOR pathway are shown in the heatmap (Figure S2A). Furthermore, the effects of *Supt16* haploinsufficiency on the expression of gene sets related to the autophagy process in hNSCs were examined by GSEA. The results showed that *Supt16* haploinsufficiency led to an overactive autophagy process in hNSCs (Figure 2E); the relevant genes are shown in the heatmap (Figure S2B). According to the above gene expression pattern analysis results, we found that *Supt16* haploinsufficiency raised the autophagy process by inhibiting the PI3K/AKT/mTOR pathway. To further investigate the effects of *Supt16* haploinsufficiency on the PI3K/AKT/mTOR/autophagy pathway, we analyzed the expression levels of related proteins. Western blot analysis showed that the AKT expression level had no significant change (Figure 2F,G), whereas the reduced levels of phospho-AKT and phospho-mTOR were observed in *Supt16* heterozygous hNSCs (Figure 2F–H). For mTOR, a vital regulator of autophagy and whose activation degree is closely related to the autophagy level, we analyzed the level of autophagy in *Supt16*^+/−^ hNSCs. Notably, western blot analysis exhibited that *Supt16* haploinsufficiency increases the protein levels of autophagy marker LC3 and promotes cytoplasmic LC3 I transfer into the autophagosome membrane protein LC3II (Figure 2F,I). Meanwhile, with the level of autophagy increased, *Supt16* heterozygous hNSCs showed reduced PAX6 and SOX2 and increased P53 protein expression compared to the control group (Figure S2C,D). Together, these studies indicated *Supt16* was functional in hNSC self-renewal and its haploinsufficiency affected the self-renewal ability of hNSCs by regulating the PI3K/AKT/mTOR/autophagy pathway.

### 2.3. Wild-Type hNSCs Treated by Rapamycin Reproduced the Phenotype of Supt16 Haploinsufficient hNSCs

**Figure 3:**
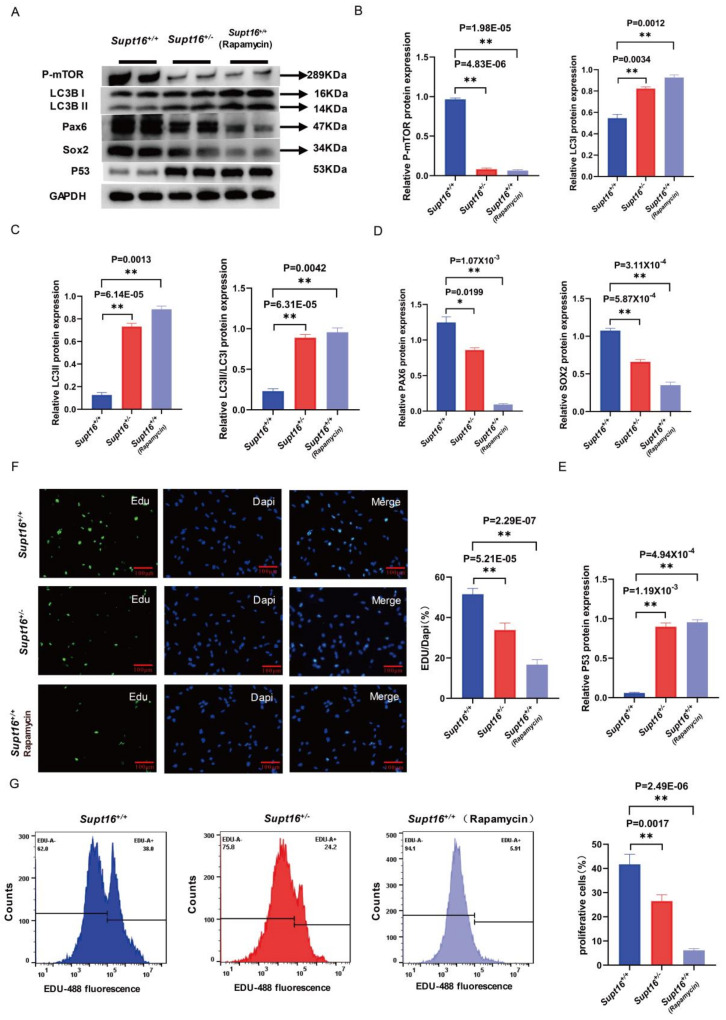
Wild−type hNSCs treated by rapamycin reproduced the phenotype of *Supt16* haploinsufficient hNSCs. (**A**) Western blot analysis showing P−mTOR, LC3, PAX6, SOX2, and P53 protein expression in hNSCs treated with different treatments (*n* = 3). (**B**) Quantification analysis of P−mTOR, LC3I protein relative to GAPDH. Data are means ± standard deviation of three independent experiments. (**C**) Quantification analysis of LC3II and LC3II/LC3I protein relative to GAPDH. Data are means ± standard deviation of three independent experiments. (**D**) Quantification analysis of PAX6 and SOX2 proteins relative to GAPDH. Data are means ± standard deviation of three independent experiments. (**E**) Quantification of P53 protein relative to GAPDH. Data are means ± standard deviation of three independent experiments. (**F**) Immunofluorescence staining showed that the absence of *Supt16* in hNSCs suppressed the proliferation. Rapamycin treatment reproduced the inhibitory effect of *Supt16* haploinsufficiency on the proliferation of hNSCs. (**G**) Cell cycle analysis using PI staining in WT, *Supt16* heterozygous and rapamycin-treated WT hNSCs (*n* = 3). The different cell cycle phases were calculated in FlowJo (v10.4.0). The error bars represented the mean ± SD and the significance level was calculated by Student’s t-test (two-tailed, equal variance) (ns means not statistically significant, * *p* < 0.05, ** *p* < 0.01).

Transcriptome analysis revealed that the impaired self-renewal ability of *Supt16*^+/−^ hNSCs was accompanied by the activation of autophagy regulated by PI3K/AKT/mTOR suppression. To further investigate the relationship between the activation of autophagy and hNSC self-renewal, we treated wild-type hNSCs with rapamycin, a potent and specific mTOR pathway inhibitor, and an autophagy activator, to simulate the effects of hyperactive autophagy induced by *Supt16* haploinsufficiency on hNSCs. It was found that phospho-mTOR level was significantly decreased and the protein levels of LC3I, LC3 II, and LC3 II/LC3 I were increased in hNSCs treated with rapamycin (Figure 3A–C), indicating that the autophagy process was activated by the suppression of the mTOR signaling pathway in *Supt16*^+/−^ hNSCs. Simultaneously, we found that elevation of the autophagy process resulted in lower expression of SOX2 and PAX6 proteins in rapamycin-treated hNSCs compared to the control group (Figure 3A,D). Meanwhile, the notable increment of P53 protein associated with cell apoptosis was also observed in hNSCs following rapamycin treatment (Figure 3A,E). These findings were similar to the effects of *Supt16* haploinsufficiency on hNSCs (Figure 3A). Moreover, to further confirm the effects of rapamycin on the proliferation ability of hNSCs at the cellular level, the EdU incorporation assay was conducted to evaluate the proliferation ability of hNSCs. Consistent with the proliferation analysis of *Supt16*^+/−^ hNSCs, the investigation showed that the proliferation of hNSCs treated by rapamycin was decreased with the low EdU labeling rate (Figure 3F). Meanwhile, flow cytometry using EdU-488 staining was performed to further understand the extent to which rapamycin disrupted the proliferative ability of hNSCs. The results showed that rapamycin treatment and *Supt16* haploinsufficiency had similar inhibitory effects on the proliferation of hNSCs (Figure 3G). Taken together, these results proved that autophagy activation induced by the mTOR pathway in *Supt16* haploinsufficient hNSCs disrupted the self-renewal ability of hNSCs by inhibiting proliferation and promoting apoptosis.

### 2.4. MHY1485 Treatment Rescued the Phenotypes of Supt16 Haploinsufficient hNSCs

**Figure 4:**
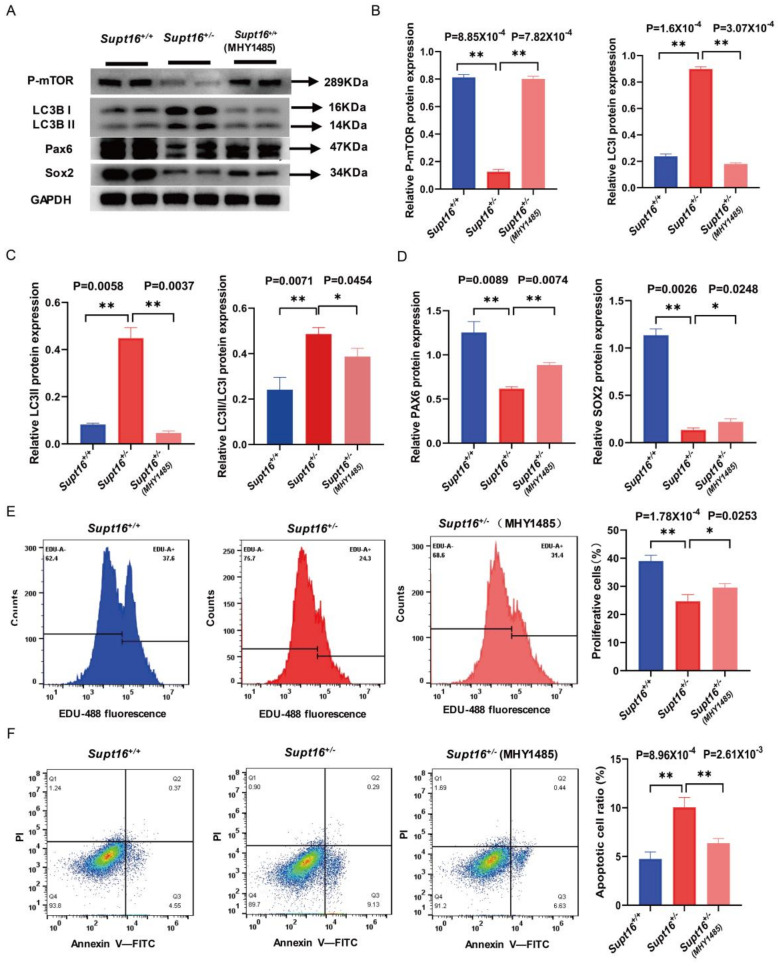
MHY1485 treatment rescued the phenotypes of *Supt16* haploinsufficient hNSCs. (**A**) Western blot analysis showed the protein expressions of P−mTOR, LC3, PAX6, and SOX2 in hNSCs treated with different treatments. (**B**) Representative quantification analysis of P−mTOR and LC3I in different hNSCs. (**C**) Quantification analysis of LC3II and LC3II/LC3I in different hNSCs. Data are means ± standard deviation of three independent experiments. (**D**) Quantification analysis of PAX6 and SOX2 in different hNSCs. Data are means ± standard deviation of three independent experiments. (**E**) Cell cycle analysis performed by flow cytometry to calculate the distribution of cell cycle phase (*n* = 3). (**F**) Flow cytometric analysis using PI and Annexin−V double staining showed the cell apoptotic rate in hNSCs treated with different treatments (*n* = 3). The error bars represented the mean ± SD and the significance level was calculated by Student’s t-test (two-tailed, equal variance) (ns means not statistically significant, * *p* < 0.05, ** *p* < 0.01).

To comprehensively investigate the role of autophagy in hNSC self-renewal capacity, we suppressed the autophagy process with MHY1485, which is a potent cell-permeable mTOR activator that targets the ATP domain of mTOR and inhibits autophagy by suppression of fusion between autophagosomes and lysosomes, to suppress autophagy process. The results demonstrated that MHY1485 treatment saved phospho-mTOR protein expression while inhibiting autophagy with lower levels of LC3 II and LC3 II/LC3 I protein in *Supt16*^+/−^ hNSCs (Figure 4A–C). Moreover, the expression of PAX6 and SOX2 proteins was elevated in *Supt16* haploinsufficient hNSC treatment with MHY1485, indicating that the impaired proliferation ability caused by autophagy in *Supt16*^+/−^ hNSCs was rescued by MHY1485 (Figure 4A,D). This finding was confirmed by the EdU incorporation assay, which found that MHY1485 treatment rescued the proportion of EdU-labeled proliferative cells in *Supt16*^+/−^ hNSCs (Figure S3A). Meanwhile, flow cytometry analysis showed that MHY1485 could partially rescue the proliferation ability of *Supt16* heterozygous hNSCs (Figure 4E). Furthermore, cell apoptosis analysis by flow cytometry revealed that the suppression of autophagy induced by MHY1485 diminishes apoptosis in *Supt16*^+/−^ hNSCs (Figure 4F). Taken together, these data demonstrate that inhibition of autophagy by MHY1485 could rescue the self-renewal ability by suppressing apoptosis and promoting proliferation in *Supt16*^+/−^ hNSCs.

## 3. Discussion

Many studies indicated that the proliferation defect in NSCs is a major convergence point for NDDs [7]. The *Supt16* gene encodes the large subunit of facilitates chromatin transcription (FACT) consisting of subunits SPT16 and SSRP1. Which is highly expressed in proliferating cells and hardly expressed in terminally differentiated cells [37]. Previous studies showed that *Supt16* directly regulates Wnt signaling pathway activity [31]. Moreover, SPT16 and OCT4 could together promote the expression of stemness genes in mouse embryonic stem cells [32]. Furthermore, Jonay Garcia-Luis et al. found that FACT interacts directly with cohesion and participates in cell cycle regulation [38]. Mengqi Ma et al. also revealed that *dre4*, the fly ortholog of *Supt16H* in *drosophila*, is required for proper cell growth and survival in multiple tissues [39]. More interestingly, de novo missense variants in *Supt16H* that cause intellectual disability, autistic features, minor dysmorphic features, and seizures have been identified in patients with NDDs [33]. Our previous research echoed these results, revealing that *Supt16* haploinsufficiency leads to impaired mNSCs proliferation, which in turn leads to NDD phenotypes caused by the reduction of neurons in mice. This was the first article to study the pathogenic mechanism of NDDs caused by the *Supt16* gene [34]. However, none of these studies elucidated the effects of *Supt16* on hNSC self-renewal ability. Our investigation demonstrated that *Supt16* haploinsufficiency damaged the self-renewal of hNSCs by activating the autophagy process governed by the suppression of the PI3K/AKT/mTOR signaling pathway. This is the first paper to show that *Supt16* plays an essential role in hNSC self-renewal and provides further evidence that the *Supt16* gene is crucial for human development.

Similar to the phenotypes of NSCs in *Supt16* heterozygous mice [34], *Supt16* haploinsufficient hNSCs also exhibited defective proliferation, cell cycle arrest in the G1/S phase, and increased apoptotic rate. Mechanistically, in contrast to the inhibition of the MAPK pathway in mNSCs, we found that *Supt16* haploinsufficiency raised autophagy by inhibiting the PI3K/AKT/mTOR pathway in hNSCs. These findings revealed differences between mNSCs and hNSCs in the underlying mechanisms by which *Supt16* haploinsufficiency affects neural stem cell function, suggesting that *Supt16* haploinsufficiency might result in similar phenotypes through different mechanisms due to species differences. Furthermore, it is worth noting that the investigations from animal models cannot be fully extrapolated to humans. Therefore, it is of great significance for us to use the iPSCs model to further explore the effects and underlying mechanisms of *Supt16* haploinsufficiency on hNSCs, which is crucial for fully revealing the role of *Supt16* in human neural stem cell self-renewal. It is also an essential implication for latecomers to study human-related diseases in animal models. Moreover, this study further advances the understanding of the relationship between *Supt16* and human NDDs.

Autophagy is a process in which the cell self-digests its own components. This self-digestion not only provides nutrients to maintain vital cellular functions but also can rid the cell of superfluous or damaged organelles, misfolded proteins, and invading microorganisms, essential for survival, differentiation, and development [40]. The lysosomal degradation pathway of autophagy plays a fundamental role in cellular, tissue, and organismal homeostasis and is mediated by evolutionarily conserved autophagy-related (ATG) genes [20]. Recent human genetic and genomic evidence has demonstrated the emerging, significant role of autophagy in human brain development and prevention of a spectrum of NDDs [21]. In recent years, accumulating evidence indicates that autophagy is required for stem cell quality control [41,42]. Defective autophagy results in a progressive loss of NSCs and impairment in neuronal differentiation specifically in the postnatal brain in mice [43]. Autophagy deficiency also suppresses adult hippocampal neurogenesis in mice by inducing the autophagic cell death of hippocampal NSCs [44]. Growing evidence has shown that autophagy plays a significant role in human brain development and improper autophagy leads to a variety of NDDs [22,23,24]. Similar results were found when *Supt16* was heterozygous; the level of autophagy gradually increased with the heterozygous knockout of the *Supt16* gene and led to the reduced expression of PAX6 and SOX2 and increased P53 protein, which eventually impaired the self-renewal ability of hNSCs by inhibiting proliferation and promoting apoptosis. Further confirming many previous reports on NDDs caused by autophagy defects, our study showed that abnormal activation of autophagy is detrimental to neural development. Interestingly, the impaired self-renewal ability in *Supt16*^+/−^ hNSCs could be reproduced by the autophagy activator rapamycin in wild-type hNSCs and reversed by the autophagy inhibitor MHY1485. This finding suggests that inhibition of autophagy with appropriate autophagy inhibitor doses is a possible therapeutic option in NDDs, due to excessive activation of autophagy. Thus, we indicate that autophagy may be a possible target for the therapy of NDDs in the clinic, based on our research and earlier studies utilizing rapamycin to correct the phenotypes of those conditions resulting from defective autophagy.

## 4. Method

### 4.1. Generation of Supt16^+/−^ hNSCs

The PGP1 cell line was obtained from the Department of Cardiology, Boston Children’s Hospital, Boston, MA, USA (Gift from George Church, available at NIGMS Human Genetic Cell Repository/Coriell #GM23338). *Supt16*^+/−^ iPSCs were generated by CRISPR/Cas9 technology [45]. Briefly, we designed a gRNAs target on exon 1 of *Supt16* (using the website https://portals.broadinstitute.org/gppx/crispick/public accessed on 1 March 2022). The gRNA sequences are: gRNA1: 5′-AGAGACTGTACAGCAATTGG-3′; gRNA2: 5′-TGTACAGCAATTGGCGGGTG-3′. gRNAs were cloned into the px458-U6expression vector with EGFP (https://www.addgene.org/ accessed on 1 March 2022). The plasmids encoding gRNA and hCas9 were co-transfected into PGP1 using basic Nucleofector kit prim (Lonza, VAPI-1005, Basel, Switzerland). The cells were then planted on a six-well plate coated with 0.1% gelatin to form colonies. When the convergence degree of cells reached about 80%, we selected monoclonal cells and extracted DNA to identify the genotype of monoclonal cells primer (forward, 5’- CCGTCCATCCGAAAG AGGGTT-3’) (reverse, 5’-GAAAAGGGTGAGGCACAGAAC-3’). After analysis, we found an insertion of a base (A) in the first exon of the *Supt16* gene (g.157_158ins A).

### 4.2. Human Neural Stem Cell Differentiation

The human iPSCs were differentiated into hNSCs with the protocol provided by CeLLapy (https://www.cellapybio.com/productinfo/1804593.html accessed on 1 March 2022). We grew iPSCs to 100% confluence in a six-well plate, then the human neural stem cell induction medium was removed from the refrigerator and equilibrated to room temperature. Next, the iPSCs medium was discarded, and the neural stem cell induction medium was added to six-well plates after washing three times with PBS. The iPSCs were cultured in a constant temperature cell incubator at 37 °C with 5% CO_2_. Every 48 h, the induction medium was completely changed, and the human neural stem cell induction medium (6 mL/well) was changed each time. For neural stem cells differentiation, iPSCs were cultured for six days using the neural induction medium. Under the microscope, a large number of scattered rosettes appeared, which was the end of the induction phase (Figure S4A). The iPSCs were induced to differentiate for about six days to obtain rosettes, which were then digested into single cells using accutase (SIGMA Technology, 3050 Spruce Street, Saint Louis, MO, USA, A6964). The hNSCs were then cultured in the maintenance medium (Human) (Cat#05750) containing rhbFGF (Stem cell, 78003, Vancouver, BC, Canada), heparin solution (Stem cell, 07980), EGF (Gibco, PMG8041, Waltham, MA, USA), and NeuroCultTM NS-A Proliferation Supplement (Human) (Cat#05753). The cells were first cultured for 24 h to restore cell viability. Next, experiments were performed after 24 h of rapamycin or MHY1485 treatment and control cells were also cultured for 24 h accordingly.

### 4.3. Flow Cytometry Analysis Experiments

For cell cycle detection, a cell cycle detection kit (Beyotime, C1052, Suzhou, China) was used. Briefly, hNSCs were dissociated into single cells. The single cells were cultured for 24 h before the experiment and about 2 million cells were fixed overnight in 75% ethanol at 4 °C. After the cells were washed 3 times with cold D-PBS, propidium iodide (PI) was added and incubated at room temperature for 30 min in the dark. Finally, these cells were analyzed using a BDACCURIC6P flow cytometer and the PI fluorescence was detected through PE channel.

The Anaixin V-FITC/PI Cell Apoptosis Detection Kit (Beyotime, C1062M) was used for cell apoptosis detection. About 1 million cells were resuspended with cold D-PBS. Afterwards, the cells were suspended for 15 min at 37 °C in combined buffer supplemented with Annexin V-FITC and PI. Finally, these cells were analyzed using a BDACCURIC6P flow cytometer. It is worth noting that the PI fluorescence was detected through PE channel and the Annexin-V through FITC channel.

### 4.4. EdU Proliferation Assay

To study cell proliferation at the cellular level, EdU incorporation assay with a KFlour488 Click-iT EdU imaging detection kit (BeyoClick™ EdU-488, Cat# C0071S) was performed according to the instructions. Briefly, EdU experiments were performed after 24 h of single cell culture; hNSCs were incubated with 10 µM EdU on the chamber slide for 2 h. Next, the sample was washed with cold D-PBS twice. After that, the cells were fixed with 2 mL 4% paraformaldehyde solution for 5 min, followed by glycine buffer for neutralization. This was repeated three times. Then, the cells were incubated with 3 mL 0.1% Triton-100 in cold PBS for 5 min each time. After that, they were processed with EdU-KFlour488 Click in the dark for 30 min. Then, the DAPI was used to stain the nuclei of hNSCs for 10 min. After that, they were washed three times with cold PBS for 15 min. The glass slides were mounted on histological slides with glycerol. Finally, pictures were taken with a Leica DMI8 fluorescence microscope (Wetzlar, Germany) and measured by ImageJ software. The fluorescence intensity was evaluated based on the IF images from at least three physiologically separate experiments. The chosen area was used to calculate the fluorescence intensity for both the *Supt16*^+/−^ group and the control group. At least three images from each group were evaluated and a mean fluorescence intensity value (after subtracting background) was calculated. The mean fluorescence intensity value of the control group was then set as 1 (or 100%). The two-tailed unpaired t-test was used to obtain the p-values for the data.

### 4.5. Western Blot

To extract proteins, hNSCs were collected and lysed in PierceTM RIPA buffer (Thermo, 78510, Waltham, MA, USA) (100X Phosphatase Inhibitor Cocktail, GRF101.100X Protease Inhibitor Cocktail, GRF102). The Bradford method was used to quantify the lysates before equal amounts of proteins were added for the western blot assay. Antibodies used for the western blot were anti-SPT16 (Abcam, ab204343, 1:2000, Cambridge, UK), anti-PCNA (Abcam, ab92552, 1:2000), anti-phospho-mTOR (Ser2448) (CST, Cat#5536T, 1:2000), anti-phospho-AKT (S473) (CST, Cat#4060S, 1:1000), anti-AKT (CST, Cat#4691S, 1:2000), anti-PAX6 (Abcam, ab195045, 1:2000), anti-LC3B (Abcam, ab192890, 1:2000), and anti-SOX2 (Abcam, ab92494, 1:2000). Before being transferred to a PVDF membrane, the proteins were separated using 12% SDS-PAGE (BIO-RAD, TGX Stain-Free Fast Cast Acrylamide Kit 12% Cat#1610185) for 2 h. After incubation with QuickBlock^TM^ (Beyotime, P0252) for 30 min at room temperature. The primary antibodies were incubated with the membrane at 4 °C for the whole night. The membranes were then subjected for two hours at room temperature to HRP-conjugated goat anti-rabbit IgG (GtxRb-003-DHRPX, immunoReagents, 1:5000). At least three different western blot studies were conducted. Using ImageJ software (https://imagej.nih.gov/ij/docs/guide/146-30.html accessed on 1 March 2022), the western blot bands were quantified.

### 4.6. RNA-Seq Analysis

Total RNA was obtained by extracting hNSCs derived from iPSCs using a Total RNA kit (Omega, R6834-01, Biel/Bienne, Switzerland). Using the PrimeScriptTMRT reagent kit with gDNA Eraser (Perfect Real Time) (TaKaRa, Cat#RR047A, Kusatsu, Japan), 1 g of total RNA was reverse transcribed into cDNA. The TB Green Premix TaqTM II (TIi RnaseH Plus) (Cat#RR820A) was used to conduct RT-qPCR. For necessary purposes, the author might be contacted to receive the RT-qPCR primers. The experiments were conducted three times. Based on the 2^-ΔΔCt^ technique, relative gene expression levels were calculated. Data were shown as means and SEM. The two-tailed unpaired t-test was used to obtain *p*-values.

Trizol was used for hNSCs generated from iPSCs for RNA-seq (Invitrogen, Waltham, MA, USA). They were then sent to Novogene in Beijing, China (https://cn.novogene.com accessed on 1 March 2022) for deep sequencing and building RNA-seq libraries. The sequences were aligned to the Homo Sapiens (GRCh38/hg38) (Index of/pub/release-108/fasta/homo_sapiens/dna (ensembl.org) reference genomes. Functional enrichment analysis and differential gene expression were performed using DESeq223 and GSEA24, respectively. Studies in downstream bioinformatics and statistics were also completed in the R environment. Three biological repeat sequences were discovered. To find DEGs, the criteria FDR < 0.05 and a Log2 fold change > 1 were applied.

## 5. Conclusions

Taken together, our results indicated that *Supt16* haploinsufficiency disrupts hNSC self-renewal. They also showed that the overactive autophagy regulated by PI3K/AKT/mTOR pathway inhibition was responsible for the phenotype of impaired self-renewal, fully indicating the important role of autophagy in stem cell self-renewal, as shown in previous studies [46].

## Data Availability

Data generated for this article will be obtained from the corresponding author upon reasonable request.

## Supplementary Materials

The following supporting information can be downloaded at: https://www.mdpi.com/article/10.3390/ijms24033035/s1.
