# Supplementary material for: Supt16 Haploinsufficiency Impairs PI3K/AKT/mTOR/Autophagy Pathway in Human Pluripotent Stem Cells Derived Neural Stem Cells
Source: Int J Mol Sci. 2023 Feb 3;24(3):3035. doi: 10.3390/ijms24033035 (PMC9917370; doi:10.3390/ijms24033035)
Supplement: Supplementary file 1 [file ijms-24-03035-s001.zip › ijms-2155869-supplementary.pdf]

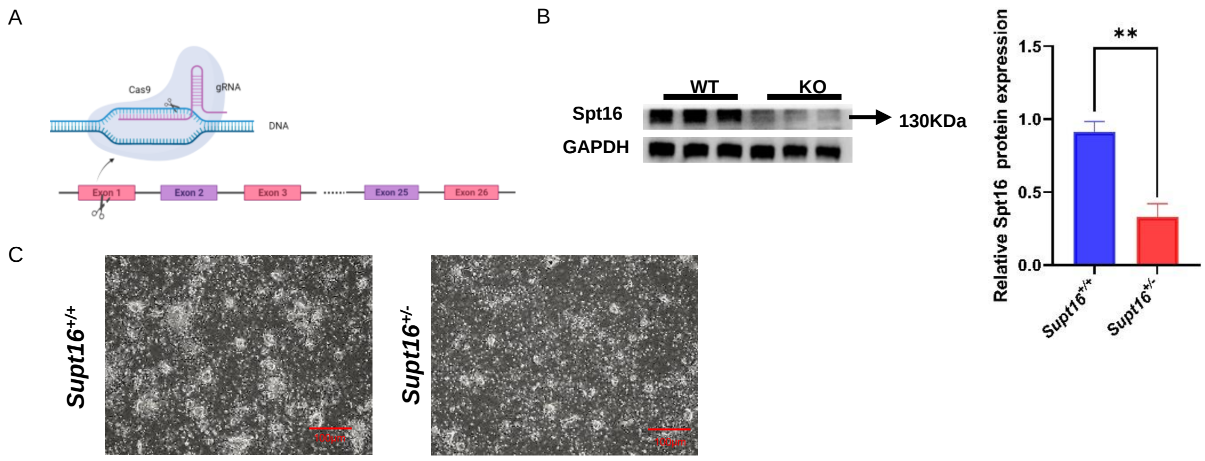

Figure S1. A; Schematic representation of CRISPR/Cas9 gRNA editing sites in exon 1 of *Supt16.* B; Western blot and quantification of Spt16 protein level in hNSCs (n = 3). Data are means $\pm$ standard deviation of three independent experiments.
C; Representative images of human neural stem cells.

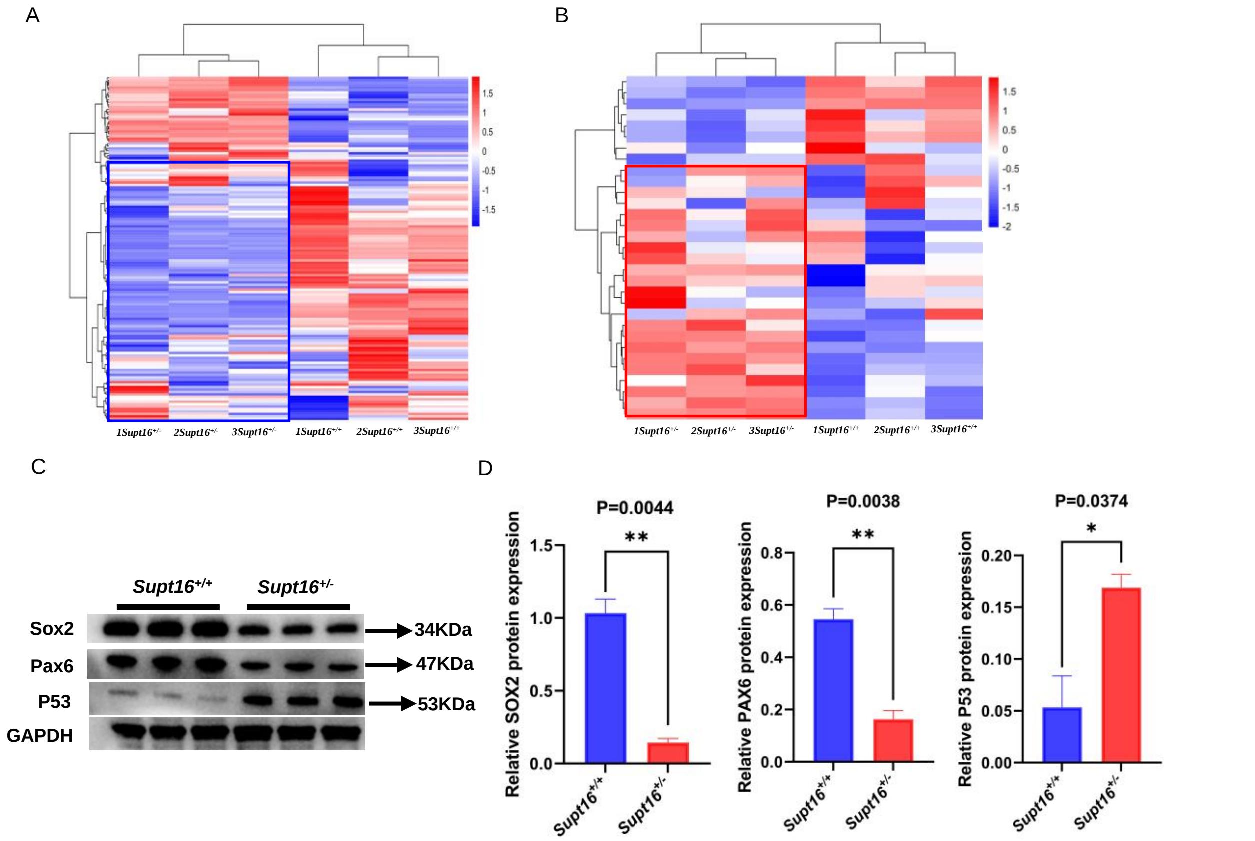

Figure S2. A; Heatmap showing differential expression genes of mTOR signaling pathway enriched in GSEA analysis. B; Heatmap showing autophagy-related differential expression genes analyzed by GSEA.
C and D; Western blot and quantification of PAX6, SOX2 and P53 protein level in hNSCs (n = 3). Data are means $\pm$ standard deviation of three independent experiments.

A

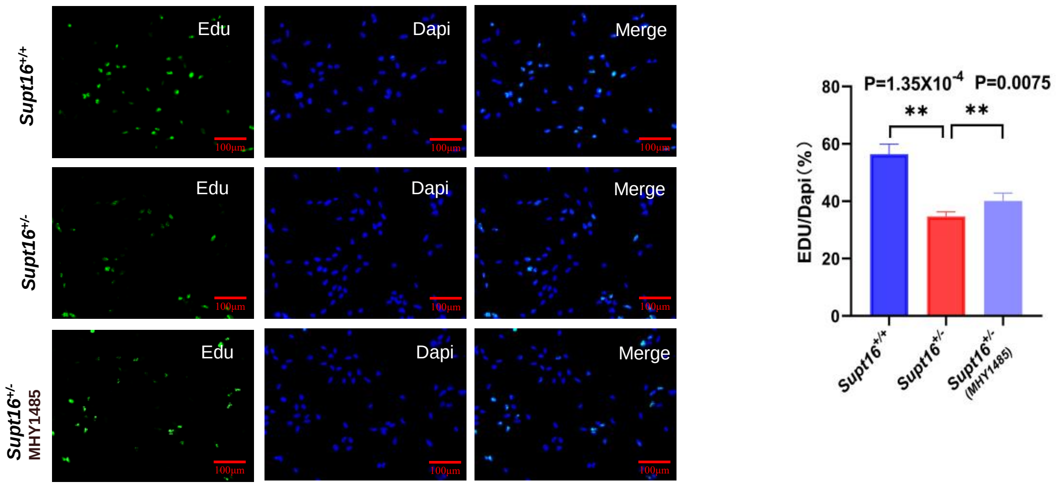

Figure S3. A; Representative images and quantification of EDU (green) immunofluorescence staining in hNSCs treated with different treatments. And MHY1485 treatment rescued the inhibitory effect of *Supt16* haploinsufficiency on the proliferation of hNSCs.

A

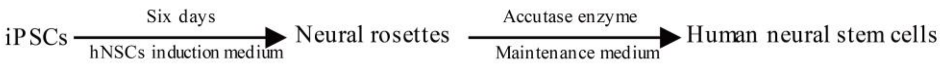

Figure S4. A; Schematic diagram of the human neural stem cell differentiation process.
